# Development and the initial validation of a new self-administered questionnaire for an early detection of health status changes in smokers at risk for chronic obstructive pulmonary disease (MARKO questionnaire)

**DOI:** 10.3325/cmj.2016.57.425

**Published:** 2016-10

**Authors:** Žarko Vrbica, Marina Labor, Adrijana Košćec Đuknić, Biserka Radošević-Vidaček, Ivan Gudelj, Slavica Labor, Iva Jurić, Peter MA Calverley, Davor Plavec, for the MARKO study group

**Affiliations:** 1 Department of Pulmonology and Immunology, General Hospital Dubrovnik, Dubrovnik, Croatia; 2 University of Dubrovnik, Dubrovnik, Croatia; 3 Department of Pulmonology, University Hospital Center Osijek, Osijek, Croatia; 4 Faculty of Medicine, J.J. Strossmayer University of Osijek, Osijek, Croatia; 5 University of Zagreb Centre for Croatian Studies, Borongaj Campus, Zagreb, Croatia; 6 Institute for Medical Research and Occupational Health, Zagreb, Croatia; 7 Department of Pulmonology, University Hospital Center Split, Split, Croatia; 8 Department of Internal Medicine, University Hospital Center Osijek, Osijek, Croatia; 9 University of Liverpool, Liverpool, UK; 10 Research Department, Children’s Hospital Srebrnjak, Zagreb, Croatia; * The first two authors contributed equally.

## Abstract

**Aim:**

To develop and do an initial validation of a new simple tool (self-administered questionnaire) that would be sensitive and specific enough to detect early changes in smokers leading to future development of chronic obstructive pulmonary disease (COPD).

**Methods:**

224 consecutive participants (50.9% women), with mean ± standard deviation age of 52.3 ± 6.7 years, 37.5 ± 16.7 pack-years smoking history (85.8% active smokers), and no prior diagnosis of COPD were recruited. The MARKO questionnaire was self-administered twice; at the general practitioner's office and after 2-4 weeks at the tertiary care hospital. Participants were assessed for COPD by a pulmonologist after filling in a quality of life (QoL) questionnaires, history-taking, physical examination, lung function test, 6-minute walk test, and laboratory tests. They were divided into four subgroups: “healthy” smokers, symptomatic smokers, and smokers with mild and moderately severe COPD.

**Results:**

Psychometric analyses indicated that the 18-item questionnaire had a very good internal consistency (Cronbach’s alpha = 0.91) and test-retest reliability for a four week period (ρc = 0.89, 95% confidence interval [CI] 0.85-0.92, Lin’s concordance). A significant correlations of MARKO scores were found with two QoL questionnaires; r = 0.69 (*P* < 0.001) and r = 0.81 (*P* < 0.001). Receiver operating characteristic curve analysis showed an area under the curve of 0.753 (95% CI 0.691-0.808, *P* < 0.001), with a sensitivity of 71.83% and specificity of 64.24% to discriminate “healthy” smokers from other subgroups.

**Conclusion:**

Based on psychometric analyses and high convergent validity correlation with already validated QoL questionnaires, the newly developed MARKO questionnaire was shown to be a reliable self-administered short health status assessment tool.

**Trial registration:**

Clinicaltrial.gov NCT01550679

Chronic obstructive pulmonary disease (COPD) is one of the major causes of chronic morbidity and mortality throughout the world (1). Millions of people suffer from this disease for years, and die prematurely from it or its complications, thus producing a significant impact on the health care system and economy. Since COPD is a preventable and treatable disease, early detection is very important (1,2). Chronic airflow limitation, which is a major characteristic of COPD, is caused by a mixture of small airways lesions and parenchyma destruction caused by chronic inflammation. According to the Global Initiative for Chronic Obstructive Lung Disease (GOLD), to establish the diagnosis, the patient has to have a significant exposure, characteristic symptoms, and a significant degree of airflow limitation (Tiffeneau index <0.7) (1). American Thoracic Society/European Respiratory Society (ATS/ERS) use even more stringent criteria for a significant airflow limitation based on the lower limit of normal (LLN), arguing that using a single point criteria for all age groups significantly under- or overestimates the incidence and prevalence of COPD in different age groups (3). On the other hand, pathophysiological changes, symptoms, and diminished health related quality of life (HRQoL) often precede clinically significant airflow limitation (1). Even though cigarette smoking is the major cause of COPD, only a fraction (<1/3) of the smokers develop the disease. Based on the recently published data from a subsample (n = 50 008) of the UK Biobank, there are significant shared genetic mechanisms underlying airway limitation, COPD, and smoking addiction (4). Despite recommendations for an early diagnosis (1,2), up until now, there have been no predictive parameters to evaluate the risk for developing COPD in a particular person exposed to tobacco smoke. Interdisciplinary Association for Research in Lung Disease (*Associazione Scientifica Interdisciplinare per lo Studio delle Malattie Respiratorie*, AIMAR) guidelines recommend using a stepwise approach that starts with the screening questionnaire as a first step in the identification of a high risk population. Already validated HRQoL questionnaires like St’ George Respiratory Questionnaire (SGRQ) or COPD Assessment Test (CAT) have not been developed and validated for such a purpose (5,6). The aim of our study was to construct, develop, and conduct an initial validation of a new simple tool (self-administered questionnaire) that would be sensitive and specific enough to detect early changes in smokers leading to future development of COPD.

## Methods

This study was a part of broader research project “Early Detection of COPD Patients in GOLD 0 (Smokers) Population – MARKO Project.” The details of the protocol of the MARKO project can be found at *https://clinicaltrials.gov/ct2/show/NCT01550679*. The study was approved by the Children’s Hospital Srebrnjak Ethics Committee and conducted according to the most recent version of the Declaration of Helsinki, Good Clinical Practice, and other relevant international and national laws. All participants signed the Informed consent before starting any procedure related to the study.

### Pilot study for understanding/comprehension

A cross-sectional study was conducted in 2009 on a primary care level in a large city (Zagreb and surroundings) to assess the prevalence of COPD. A subgroup of 138 patients of both sexes was chosen based on a previously diagnosed COPD for this pilot study. The study was undertaken through 17 general practitioners’ (GP) offices that possessed the COPD-6^TM^ pocket screening spirometer (4000 COPD-6^TM^ Respiratory Monitor, Vitalograph Ltd, Buckingham, UK).

The GPs were asked to collect the data on all the patients who were active smokers with ≥20 pack-years smoking history and over 40 years of age, irrespective of the reason of their visit, about their chronic respiratory conditions including COPD, asthma, or any other respiratory condition, respiratory therapy, and exacerbations during the past year. They were also asked to measure lung function using the COPD-6^TM^ (forced expiratory volume in 1 second [FEV_1_], forced expiratory volume in 6 seconds [FEV_6_] as a surrogate measure for forced vital capacity [FVC], FEV_1_/FEV_6_ ratio, and lung age). Patients (n = 138) with COPD assessed the readability and comprehension of the MARKO questionnaire items. Each of the 18 items in MARKO questionnaire was rated on a scale from 1-4 (1 meaning lowest level of understanding/comprehension and 4 meaning the complete understanding/comprehension of each item) (Supplementary material)(web extra material 1).

### Validation study

*Participants.* The study conducted between 2010-2013 included 224 consecutive participants (50.9% women) with mean ± standard deviation age of 52.3 ± 6.7 years and 37.5 ± 16.7 pack-years smoking history (85.8% active smokers). They were recruited at 15 GP offices representing an equal number of GPs in two major cities (Zagreb and surroundings 7 GPs, Split and surroundings 8 GPs). The participants were approached by their GPs during a random visit to their office (not related to respiratory problems) if they were smokers or ex-smokers of the predefined age group. The pre-screening for inclusion/exclusion criteria was conducted through a structured interview. The inclusion criteria were that participants have to have signed the written consent; be smokers/ex-smokers of either sex aged 40-65 years with a smoking history of at least 20 pack-years (calculated as the number of cigarettes smoked per day multiplied by the number of years of smoking divided by 20); and have no previous diagnosis of COPD. The exclusion criteria were any clinically relevant chronic disease (cardiovascular, cerebrovascular, diabetes, hepatitis, nephropathy, chronic dialysis, systemic disorder, cancer) significantly affecting QoL at the time of the first visit; immunosuppressive therapy; preceding acute respiratory disease four weeks before the visit; hospitalization for any reason during past three months; myocardial infarction, cerebrovascular infarction or transient ischemic attack during past six months; diagnosis of asthma; and an inability to perform the diagnostic protocol. After the diagnostic workup participants were divided into four subgroups defined as “healthy” smokers (no respiratory symptoms and FEV_1_/FVC≥0.7, n = 72), symptomatic smokers (chronic respiratory symptoms as dyspnea, cough and/or sputum production and FEV_1_/FVC≥0.7, n = 110), COPD GOLD 1 (chronic respiratory symptoms, FEV_1_/FVC≥0.7 and FEV_1_≥80% predicted, n = 23), and COPD GOLD 2 (chronic respiratory symptoms, FEV_1_/FVC≥0.7 and FEV_1_<80% and ≥50% predicted, n = 19) (1).

### Measuring instruments

The MARKO questionnaire was constructed and developed in the Croatian language for the purpose of this study by a group of experts; three medical doctors (pulmonologists ŽV, DP, and PMAC) and two psychologists (BRV, AKĐ). It was constructed in the Croatian language because the whole MARKO study was planned and performed in Croatia and did not involve participants from other countries. The questionnaire comprised 18 questions covering the manifestation and frequency of the symptoms present at the early stages of the COPD that could impact the patients’ HRQoL. The participants were asked to rate the frequency of their symptoms over a designated period of time (eg, over past three months for coughing, shortness of breath, expectoration, and over past 12 months for pulmonary infections). They also rated their breathing quality and general health status. Furthermore, they reported on the shortness of breath during daily life activities requiring different physical strain, and compared their physical abilities and fatigue with respect to their referential age group. The total scores ranged from 0 to 57 points, with the higher scores indicating poorer HRQoL.

CAT is a validated, short (8-item), and simple self-administered questionnaire, with good discriminant properties, developed for use in routine clinical practice to measure the health status of patients with COPD (6). The test was developed using Rasch analysis as a single dimensional construct. Internal consistency was excellent with Cronbach's α = 0.88 and a good test-retest reliability (intraclass correlation coefficient = 0.8). Every item is rated on a six point scale from 0 to 5. Total scores range from 0 (indicating no impairment) to 40 (indicating maximum impairment). It is openly accessible and available in more than 60 languages. It was validated in 6 different countries using 4 different languages and translated to the Croatian language using an internationally recommended procedure (7).

SGRQ was designed to measure the overall health status and well-being of the patients with obstructive airways disease (5). It is a standardized self-administered airways disease-specific questionnaire divided into three domains: symptoms (8 items), activity (16 items), and impacts (26 items). Internal consistency (Cronbach's α) for these domains for COPD was 0.61, 0.90, and 0.88, respectively. For each domain and for the overall questionnaire, scores range from zero (no impairment) to 100 (maximum impairment). The questionnaire is available in more than 70 languages and openly accessible. SGRQ was not previously validated for the Croatian language but was translated using an international recommended procedure and used widely in many COPD clinical trials in Croatia (7). The SGRQ scores in our study were calculated using score calculation algorithms and missing data imputation (if total number of missing items was ≤10) using the Excel® SGRQ calculator.

### Procedure

The purpose of this initial validation was to understand the basic psychometric characteristics of this newly constructed questionnaire and determine how it compares to the already existing and validated HRQoL questionnaires used for COPD, like CAT and SGRQ. Also it was important to understand if the newly developed questionnaire discriminates between all 4 subgroups of participants. We also understand that we have different domains and some redundant questions as they differ in the level of symptoms severity. They were put into the construct on purpose because the final evaluation would be made based on the results of participants’ follow-up. The main purpose why this questionnaire was developed was to try to pick-up early changes in HRQoL that are predictive for the future development of COPD in smokers at risk, or with a progression of an early COPD. As there are no up-to-date instruments that can be compared with this, the second validation will be done using follow-up data that will allow us to discard redundant questions and fully analyze the construct validity of MARKO questionnaire. The MARKO questionnaire was self-administered twice in a validation study; first at the GP’s office and after 2-4 weeks at the tertiary care hospital during pulmonologist’s assessment. During the pulmonologist’s assessment the staff and the participant were blinded for the results of MARKO questionnaire obtained at the GP’s office. At the tertiary care hospital participants were referred to a designated team consisting of a pulmonologist, study nurse, and lung function laboratory technician. They filled in the self-administered MARKO questionnaire followed by CAT and SGRQ, after which they went through a structured and predefined diagnostic workup (history-taking, physical examination, lung function with bronchodilator test, 6-minute walk test, laboratory tests) to determine the diagnosis and staging of their COPD according to the GOLD and were divided in four subgroups that were used for further comparisons as previously described (1).

### Data analyses

Data analyses were conducted using STATISTICA version 12 (StatSoft, Inc., OK, USA) and MedCalc Statistical Software version 15.8 (MedCalc Software bvba, Ostend, Belgium). Categorical data are presented as absolute numbers and percentages. Quantitative data are expressed as mean and standard deviation (SD) or median and interquartile range (IQR). Normality of distribution was assessed using Kolmogorov-Smirnov test. Metric characteristics of the MARKO questionnaire were analyzed using Cronbach’s alpha, Lin’s concordance, and Pearson or Spearman’s correlation coefficients analyzing inner consistency, test-retest reliability, and association with other measures of HRQoL and health status. Categorical data were compared between groups using χ^2^ test and continuous variables using *t* test or Kruskal-Wallis ANOVA. Discriminative power of the MARKO questionnaire was analyzed using receiver operator curve (ROC) analysis and presented with area under the curve (AUC) (with 95% confidence intervals [CI]) together with the associated criterion automatically calculated by statistical software, sensitivity, specificity, and positive (PPV) and negative predictive (NPV) values. For the main outcomes, logistic regression analysis with odds ratios (ORs) and 95% CIs for the 18-item MARKO questionnaire was calculated. *P* < 0.05 was used as significant for all analyses with correction for multiple comparisons.

## Results

### Pilot study

Results of the pilot study done in 138 COPD patients (52.1% women, 54.5 [10.7] years, 35.5 [25.5] pack-years) showed that all 18 items of the MARKO questionnaire had a comparable comprehension scores. The average score for each item was greater than 3.2 (out of maximum 4), meaning the items were easy to understand. The difference for comprehension between 18 items was not significant (Friedman ANOVA χ^2^ [N = 134, df = 17] = 27.49, *P* = 0.051).

### Validation study

Men and women were of comparable age (52.0 vs 52.6 years, *P* = 0.537) at the time of inclusion but men smoked significantly more (43.0 vs 32.2 pack-years, *P* < 0.001) and were more likely to have quit (*P* = 0.012), although most participants were current smokers (85.8%) (Table 1). More than half of the participants of both sex (men, 56.4% vs women, 56.1%, *P* = 0.840) had chronic disorders other than respiratory and almost half of all participants were on some chronic disease treatment (42.7% vs 43.9%, *P* = 0.562). Men had a significantly higher body mass index (27.5 vs 25.4 kg/m^2^, *P* < 0.001) with significantly higher systolic and diastolic blood pressure (*P* = 0.014, *P* = 0.003, respectively) and a comparable heart rate (*P* = 0.751). Chronic or recurring respiratory symptoms were present in more than 60% of participants, with cough/sputum being present in approximately half of them and wheezing in more than 20%, with no significant difference between sexes (*P* > 0.300 for all comparisons). No significant difference was found in FEV_1_ (*P* = 0.620) and FEV_1_/FVC ratio (*P* = 0.066) but men had significantly lower FVC (*P* = 0.001) (Table 1).

**Table 1:** Characteristics of participants recruited in a validation study (N = 224)*^†^

Characteristics	Total (N = 224)	Men (n = 110)	Women (n = 114)	*P*
Age (years)	52.3 ± 6.7	52.0 ± 6.9	52.6 ± 6.4	0.537
Smoking history (pack-years)	37.5 ± 16.7	43.0 ± 17.9	32.2 ± 13.6	<0.001
Ex-smokers	32 (14.2)	22 (21.2)	10 (9.0)	0.012
Comorbidities	126 (56.3)	62 (56.4)	64 (56.1)	0.840
Chronic treatment	97 (43.3)	47 (42.7)	50 (43.9)	0.562
Body mass index (kg/m^2^)	26.4 ± 4.1	27.5 ± 3.9	25.4 ± 4.1	<0.001
Systolic blood pressure (mmHg)	126 ± 15	128 ± 14	123 ± 16	0.014
Diastolic blood pressure (mmHg)	80 ± 9	82 ± 9	78 ± 9	0.003
Heart rate (min^-1^)	80 ± 12	79 ± 13	80 ± 11	0.751
Respiratory symptoms	138 (61.6)	66 (60.0)	72 (63.2)	0.627
wheezing	49 (21.9)	23 (20.9)	26 (22.8)	0.731
cough	114 (50.9)	54 (49.1)	60 (53.1)	0.55
sputum	107 (47.8)	53 (48.2)	54 (47.4)	0.903
night awakenings	16 (7.1)	10 (10.3)	6 (6.3)	0.307
chest pain	25 (11.2)	11 (10.2)	14 (12.6)	0.572
Respiratory sounds				
soft	73 (32.6)	43 (39.1)	30 (26.3)	0.027
prolonged expiration	25 (11.2)	13 (11.8)	12 (10.5)	0.949
rhonchi	30 (13.4)	15 (13.6)	15 (13.2)	0.908
Lung function (post bronchodilator)*				
FVC (% expected)	109.8 ± 16.9	106.0 ± 15.0	113.4 ± 17.9	0.001
FEV_1_ (% expected)	99.7 ± 15.2	99.1 ± 14.8	100.2 ± 15.6	0.620
FEV_1_/FVC (%)	76.0 ± 6.4	75.2 ± 6.7	76.8 ± 6.0	0.066

*FVC – forced vital capacity, FEV_1_ – forced expiratory volume in 1 second.

†All data are presented as mean±SD or as number (%). Statistical significance for between sex comparisons was tested using *t* test or χ^2^-test.

Psychometric analyses indicated that the 18-item questionnaire had a very good internal consistency (Cronbach’s alpha = 0.91) and test-retest reliability for a four week period (ρc = 0.89, 95% CI 0.85-0.92, Lin’s concordance; r = 0.89, 95% CI 0.85-0.96, *P* < 0.001, Pearson correlation).

The item-to-total correlations identified four questions whose coefficients were lower than 0.50, and the scores of this revised 14-item version of the questionnaire were also tested and compared with the scores of the 18-item questionnaire.

Internal consistency of the 14-item version was a bit better (Cronbach’s alpha = 0.94), with a comparable test-retest reliability for a four week period (ρc = 0.88, 95% CI 0.84-0.91, Lin’s concordance; r = 0.88, 95% CI 0.81-0.95, *P* < 0.001, Pearson correlation). The median (IQR) scores of the 18- and 14-item versions of the MARKO questionnaire, CAT scores, and SGRQ scores and subgroup comparisons are presented in Table 2. There were no significant differences in the scores of both versions for sex (*P* > 0.200 for both). The correlations of both scores with age were not significant (r <0.02, *P* > 0.800 for both).

**Table 2:** Scores for the MARKO questionnaire, COPD Assessment Test (CAT) and St’ George Respiratory Questionnaire (SGRQ) according to different subgroups*^†^

		MARKO questionnaire		CAT score	SGRQ scores			
		18-item	14-item		symptom	activity	impact	total
All (N = 224) Range		11 (7-18.5) 0-44	8 (3.5-13.5) 0-36	8 (4-13) 0-37	14.5 (6.3-31.8) 0-100	18.3 (6-35.5) 0-79.6	3.9 (0-12.4) 0-55.3	12.5 (4.3-21.2) 0-56.5
Sex	Men (n = 110)	10 (7-17)	7 (3-13)	8 (4-12)	16.0 (4.6-30.2)	23.2 (6-32.4)	4.2 (0-14.1)	12.9 (4.4-21.8)
	Women (n = 114)	13 (6-19)	9 (4-14)	9 (4-14)	13 (7.5-34.4)	17.4 (6-35.5)	3.7 (0-9.5)	11.3 (4.1-20)
	*P*	0.162	0.18	0.147	0.805	0.975	0.361	0.625
Subgroups after diagnostic workup	HS (n = 72)	7 (3-11)	5 (2-9)	5 (2-8)	6.3 (0-14.2)	11.2 (0-29.3)	0 (0-4)	5.2 (1.9-13.3)
	SS (n = 110)	13 (9-20)^ǁ^	9 (4-15)^ǁ^	10 (6-15)^ǁ^	19.6 (11-37.3)^ǁ^	23.4 (6-35.6)	6.7 (0-13.4)^ǁ^	14.7 (6.8-24.1)^ǁ^
	COPD GOLD 1 (n = 23)	10 (8-20)^‡^	8 (5-14)	9 (4-12)	11.4 (2.6-28)	20.4 (11.2-29.5)	5.1 (0-17.2)	12.3 (3.8-21.9)
	COPD GOLD 2 (n = 19)	18 (10-26)^ǁ^	13 (7-23)^ǁ^	11.5 (6.5-18)^§^	29.2 (15.1-38.7)^§^	23.3 (17.4-47.7)	11.4 (3-20.8)^‡^	18.3 (12.1-30.1)^§^
	*P*	<0.001	<0.001	<0.001	0.039	<0.001	<0.001	<0.001
COPD	no (n = 182)	11 (6-16)	8 (3-12)	8 (4-13)	14.1 (6.3-31.7)	17.4 (6-35.4)	3.7 (0-10.6)	11.2 (4.1-19.9)
	yes (n = 42)	14 (9-24)	10 (5.5-18.5)	9 (5-15)	22.6 (5.4-34.4)	23.3 (12.4-35.8)	7.6 (0-18)	17.2 (7.5-27)
	*P*	0.008	0.015	0.133	0.264	0.226	0.098	0.09
Smoking	ex-smokers (n = 32)	10 (6.5-19)	8.5 (4-13.5)	7 (3-12)	6.3 (0-16.6)	29.5 (11.8-35.6)	4.2 (0-12.9)	15.5 (4.8-21.7)
	active (n = 192)	11 (7-19)	8 (3-14)	8 (4-14)	16.6 (8.8-34.4)	17.4 (6-32.5)	3.9 (0-12.4)	12.5 (4.4-21.3)
	*P*	0.657	0.697	0.324	0.002	0.054	0.637	0.533
Comorbidities	no (n = 98)	10 (6-16)	7.5 (3.5-12)	8 (4-13)	14.8 (6.3-34.4)	17.4 (6-29.5)	3.8 (0-11.4)	12.6 (4.3-17.4)
	yes (n = 126)	11 (7-19)	8 (4-15)	8 (4-13)	14.1 (5.1-27.9)	23.3 (6.2-35.5)	4.2 (0-13.3)	12.3 (4.4-22)
	*P*	0.238	0.113	0.943	0.414	0.132	0.527	0.44
Chronic treatment	no (n = 127)	10 (6-17)	7 (3-12)	8 (4-12)	14.1 (6.3-31.7)	17.1 (6-29.5)	2 (0-10.2)	11 (3.8-17.4)
	yes (n = 97)	11 (8-19)	8 (5-15)	9 (5-14)	14.9 (6.3-34.2)	23.5 (11.2-35.6)	6.1 (0-14.3)	14.7 (5.8-22.4)
	*P*	0.085	0.04	0.124	0.908	0.022	0.026	0.026
Respiratory symptoms	no (n = 86)	7.5 (3-14)	6 (2-10)	5 (2-9)	6.3 (0-14.2)	11.5 (0-29.5)	0 (0-4.2)	5.2 (1.9-14.4)
	yes (n = 138)	14 (9-21)	9 (5-15)	10 (6-15)	22.5 (11.1-38)	23.3 (11.2-35.5)	7.2 (0-14.9)	15 (7.9-24.1)
	*P*	<0.001	<0.001	<0.001	<0.001	0.013	<0.001	<0.001
Wheezing	no (n = 175)	10 (6-15)	7 (3-12)	7 (4-11)	11 (2.3-22.4)	17.1 (6-29.5)	2 (0-9.5)	9 (3.8-17)
	yes (n = 49)	19 (13-25)	12 (9-18)	14 (10-18)	34.6 (22.6-45.8)	29.5 (18.5-41.3)	10.3 (4.3-18.1)	21.4 (14.1-27.4)
	*P*	<0.001	<0.001	<0.001	<0.001	<0.001	<0.001	<0.001
Chronic cough/ sputum	no (n = 110)	9 (4-14)	6 (3-10)	7 (3-11)	8.9 (0-18.3)	17.1 (6-29.5)	0 (0-7.9)	7.6 (2.5-16.3)
	yes (n = 114)	14 (10-22)	9 (5-16)	10 (6-16)	22.9 (11.1-40.5)	23.4 (11.2-35.6)	7.4 (0-15.9)	15.7 (6.9-25.4)
	*P*	<0.001	<0.001	<0.001	<0.001	0.017	<0.001	<0.001
Night awakenings	no (n = 208)	11 (6-18)	8 (4-12)	8 (4-12)	12.2 (6.3-30.2)	18.2 (6.2-35.4)	3.8 (0-10.4)	12.5 (4.4-19.4)
	yes (n = 16)	23 (12-34.5)	15 (8-28.5)	17 (13-25.5)	46.9 (22.9-64.7)	26.5 (12.2-53.6)	20.9 (12.5-36.6)	27 (21.9-45.7)
	*P*	<0.001	0.003	<0.001	<0.001	0.076	<0.001	<0.001
Chest pain	no (n = 199)	10 (6-15)	7 (3-12)	8 (4-12)	11.9 (4.4-28)	17.4 (6-29.5)	3.7 (0-10.3)	11.1 (4.1-18.2)
	yes (n = 25)	20 (15-27)	16 (11-20)	13 (9-18)	31.7 (15.3-49.4)	41.8 (23.7-53.6)	12.2 (3.6-25.7)	25.3 (15.7-36.5)
	*P*	<0.001	<0.001	<0.001	<0.001	<0.001	0.001	<0.001
Fatigue	no (n = 160)	9 (4-13)	6 (3-9)	6.5 (3-10)	11.1 (2.6-27.3)	12.2 (0-23.5)	1.8 (0-7.4)	7.7 (3.6-14.7)
	yes (n = 64)	19 (14-25)	14.5 (11-20)	13 (8-18)	21.7 (9.6-40.5)	35.8 (23.7-48)	13.7 (7.1-22.9)	23.1 (17-30.1)
	*P*	<0.001	<0.001	<0.001	<0.001	<0.001	<0.001	<0.001
Soft noise on auscultation	no (n = 151)	11 (6-16)	8 (3-12)	8 (4-13)	14.1 (6.6-27.3)	17.4 (6-29.5)	3.8 (0-11.4)	11.7 (4.5-18.6)
	yes (n = 73)	11 (6-19)	8 (4-13)	8 (4-13)	11.7 (2.3-34.4)	12.4 (0-35.8)	3.9 (0-15.4)	11.1 (3.6-26)
	*P*	0.786	0.881	0.744	0.745	0.824	0.75	0.916
Prolonged expiration	no (n = 199)	10 (6-16)	7 (3-12)	8 (4-13)	11.6 (4.5-28)	17.4 (6-35.4)	3.9 (0-11.4)	11.2 (4-19.6)
	yes (n = 25)	16 (9-25)	10 (6-21)	9.5 (5.5-14)	21.1 (10.7-34.4)	23.7 (12.4-35.3)	7.4 (0-18.7)	17.1 (5.2-27.4)
	*P*	0.007	0.008	0.194	0.076	0.226	0.122	0.08
Rhonchi	no (n = 194)	10 (6-15.5)	7 (3-12)	8 (4-12)	11.6 (3.6-27.6)	17.4 (6-35.5)	3.8 (0-10.6)	11.3 (3.8-19.6)
	yes (n = 30)	18.5 (11-25)	12 (7-19)	12.5 (5.5-17)	23.8 (12.2-42.9)	18.5 (6-35.9)	10.3 (0-18.7)	13.7 (8-27.6)
	*P*	<0.001	0.004	0.021	0.001	0.581	0.025	0.031

*HS – “healthy” smokers/ex-smokers, SS – symptomatic smokers/ex-smokers, COPD GOLD 1 – participants diagnosed with chronic obstructive pulmonary disease (COPD) with Tiffeneau index <0.7 and forced expiratory volume in 1 second (FEV_1_)>80% predicted, COPD GOLD 2 – participants diagnosed as COPD with Tiffeneau index <0.7 and FEV_1_<80% and >50% predicted.

†All data are presented as median and interquartile range (IQR) and as range for the overall scores. Statistical significance for subgroups comparisons was tested using Mann-Whitney U test for all independent variables except for 4 subgroups according to diagnosis after the diagnostic workup that was tested using Kruskal-Wallis ANOVA.

‡Significantly different from HS (post-hoc analysis): *P* < 0.05.

§Significantly different from HS (post-hoc analysis): *P* < 0.01.

ǁSignificantly different from HS (post-hoc analysis): *P* < 0.001.

We found significant moderate positive correlations of MARKO scores with CAT scores of r = 0.69 (95% CI 0.59-0.79, *P* < 0.001) and r = 0.63 (95% CI 0.53-0.74, *P* < 0.001) for the 18- and 14-item versions, respectively. Comparable significant moderate positive correlations were found for the 18- and 14-item versions with the individual domains of SGRQ (Symptom score: r = 0.69, 95% CI 0.59-0.79, *P* < 0.001 and r = 0.59, 95% CI 0.48-0.71, *P* < 0.001, respectively; Activity score: r = 0.67, 95% CI 0.57-0.78, *P* < 0.001 and r = 0.71, 95% CI 0.61-0.81, *P* < 0.001, respectively; Impact score: r = 0.68, 95% CI 0.58-0.79, *P* < 0.001 and r = 0.68, 95% CI 0.57-0.78, *P* < 0.001, respectively). Strong positive correlations were found between MARKO scores and SGRQ total score (r = 0.81, 95% CI 0.73-0.89, *P* < 0.001 for the 18-item version; r = 0.80, 95% CI 0.72-0.88, *P* < 0.001 for the 14-item version).

Although analysis of variance was significant for between group comparisons (“healthy” smokers, symptomatic smokers, COPD GOLD 1, and COPD GOLD 2) for all HRQoL questionnaires (MARKO, CAT, and SGRQ, *P* < 0.001 for all), only the 18-item MARKO questionnaire showed a significantly lower median score in “healthy” smokers compared to other three groups (M = 7 vs 13 vs 10 vs 18, *P* < 0.001, *P* = 0.045 and *P* < 0.001, respectively; Table 2). Post-hoc analysis did not show a significant difference between other three groups (*P* > 0.200 for all comparisons for all HRQoL questionnaires). ROC curve analysis showed an AUC of 0.753 (95% CI 0.691 to 0.808, *P* < 0.001) with a sensitivity of 71.83% and specificity of 64.24%, PPV 48.57%, and NPV 82.91% for the MARKO score criterion of ≤10 for “healthy” smokers. Using “healthy” smokers as the reference group, 18-item MARKO questionnaire showed an OR of 1.14 (95% CI 1.08 to 1.20) for symptomatic smokers, OR of 1.10 (95% CI 1.03 to 1.18) for COPD GOLD 1, and OR of 1.17 (95% CI 1.09 to 1.26) for COPD GOLD 2 for each additional point on the scale (*P* < 0.001 for all).

The 14-item MARKO questionnaire, CAT, and SGRQ did not show significantly different scores between “healthy” smokers and COPD GOLD 1 subgroups (*P* > 0.05 for all comparisons; Table 2). Also the 18- and 14-item MARKO questionnaires were the only that significantly discriminated COPD from non-COPD participants (M = 14 vs 11, *P* = 0.008; 10 vs 8, *P* = 0.015; Table 2). ROC curve analysis for the 18-item MARKO questionnaire showed an AUC of 0.634 (95% CI 0.567 to 0.698, *P* = 0.004), with a sensitivity of 62.50% and specificity of 49.45%, PPV 21.37%, and NPV 85.71% for the score criterion of >10 for COPD. With each additional point on the scale of the 18-item MARKO questionnaire, the odds for COPD diagnosis significantly increased by 5% (OR 1.05, 95% CI 1.01 to 1.08, *P* = 0.009). AUC for the 14-item MARKO questionnaire was 0.623 (95% CI 0.555 to 0.687, *P* = 0.010). Although the scores for other questionnaires were lower in non-COPD participants, these differences were not significant (*P* > 0.09 for all).

Active smokers were significantly different from ex smokers only in the SGRQ symptoms domain (M = 16.6 vs 6.3, *P* = 0.001; Table 2). Having a comorbidity did not produce a significantly different score on any of the used questionnaires (*P* > 0.110), but using a chronic treatment for other than respiratory disorder produced a significantly different scores for the 14-item MARKO questionnaire (M = 8 vs 7, *P* = 0.040), SGRQ total score (M = 14.7 vs 11, *P* = 0.026), SGRQ activity domain (M = 23.5 vs 17.1, *P* = 0.022), and SGRQ impact domain (6.1 vs 2, *P* = 0.026; Table 2).

All four questionnaires significantly discriminated (Table 2) between the subgroups with or without chronic respiratory symptoms (*P* < 0.001 for all comparisons), with or without wheezing (*P* < 0.001 for all comparisons), with or without chronic cough and sputum (*P* < 0.001 for all comparisons), with or without night awakening (*P* < 0.01 for all comparisons), with or without chest pain (*P* < 0.001 for all comparisons), with or without fatigue (*P* < 0.001 for all comparisons), and with or without rhonchi during auscultation of lungs (*P* < 0.05 for all comparisons). ROC curve analysis for the 18-item MARKO questionnaire showed an AUC of 0.873 (95% CI 0.821 to 0.914, *P* < 0.001) with a sensitivity of 100% and specificity of 47.70%, PPV 44.14%, and NPV 100% for the score criterion of >8 for fatigue. None of the questionnaires (Table 2) significantly discriminated participants with a soft noise compared to normal noise during auscultation (*P* > 0.740), but the 18- and 14-item MARKO questionnaires showed a significantly different scores for the prolonged expiration (M = 16 vs 10, *P* = 0.007; 10 vs 7, *P* = 0.008; respectively). ROC curve analysis for the 18-item MARKO questionnaire showed an AUC of 0.667 (95% CI 0.596 to 0.731, *P* = 0.004), with a sensitivity of 56.00% and specificity of 70.62%, PPV 21.21%, and NPV 91.91% for the score criterion of >14 for prolonged expiration.

## Discussion

The main result of our initial validation study was that the MARKO questionnaire showed expected properties in a setup and population of the intended use (8). It was validated for comprehension and had a very good internal consistency and test-retest reliability, with high convergent validity correlation with the already validated COPD HRQoL questionnaires (SGRQ and CAT). A very important finding was that MARKO questionnaire better detected early symptoms in smokers than the other two questionnaires, significantly discriminating symptomatic smokers/ex-smokers and COPD patients from “healthy” smokers/ex-smokers. Almost no differences were seen between the 14- and 18- item versions of the MARKO questionnaire, with a significantly better result for the 18-item version only regarding discriminating other subgroups from “healthy” smokers/ex-smokers. These results represent the first step and a prerequisite for further validation of the MARKO questionnaire regarding its predictive power as an early marker of future development of COPD (as a single marker or in combination) that can be used for screening in a primary care setting.

Population screening for COPD is not a recommended strategy but early diagnosis in a population at risk is highly recommended because of a high proportion of undiagnosed or late diagnosed COPD associated with high morbidity (1,2,9). Several approaches for use in primary care were tested but only to make an early diagnosis of the already present COPD (10). The MARKO questionnaire showed comparable results regarding the diagnostic potential for COPD in a primary care setting to the results of a meta-analysis of COPD Diagnostic Questionnaire (CDQ) by Haroon et al (10). However, rather than constructing a diagnostic questionnaire for COPD, our aim was to construct a questionnaire that could identify early changes in HRQoL in smokers leading to subsequent development of COPD. Having such an instrument could help in starting secondary prevention earlier or starting an early intervention. In regard to this aim, the MARKO questionnaire showed a higher sensitivity for early symptoms of future possible COPD than SGRQ or CAT, with high convergent validity correlation with these already validated COPD health status questionnaires. This high convergent validity correlation is also important because it shows specificity for respiratory disorders and could probably mean that it could be associated with already known features of CAT and SGRQ, showing association with many facets of COPD, like underlying inflammation, airway limitation, breathlessness, progression of disease, morbidity, and mortality (11-15). On the other hand, at least for the 18-item version, the results were not influenced by common comorbidities and concomitant treatment. In the systematic review by Haroon et al, the major risk for bias when evaluating the questionnaires and handheld flow meters for screening purposes was inadequate blinding between index tests and spirometry, which was not the case in our study (10).

Further validation is expected after a follow-up of the cohort of smokers recruited into the MARKO study, when the potential of this tool to predict future development of COPD in smokers/ex-smokers at risk for COPD will be evaluated (as a single tool or combined with other markers).

Based on basic psychometric analyses and high convergent validity correlation with already validated HRQoL questionnaires, the newly developed MARKO questionnaire was shown to be a reliable self-administered short health status assessment tool. It had a better discriminating power for early changes associated with smoking susceptibility than other two questionnaires (CAT and SGRQ), thus being in accordance with the newest recommendations as a first step in making an early diagnosis. These properties will be tested prospectively in an ongoing cohort study to evaluate the predictive power of the MARKO questionnaire to identify individuals who will develop COPD among individuals at risk.

## References

[1] Global Strategy for the Diagnosis. Management and Prevention of COPD, Global Initiative for Chronic Obstructive Lung Disease (GOLD) 2016. Available from: http://www.goldcopd.org/*.* Accessed: October 26, 2016.

[2] Nardini S, Annesi-Maesano I, Del Donno M, Delucchi M, Bettoncelli G, Lamberti V (2014). The AIMAR recommendations for early diagnosis of chronic obstructive respiratory disease based on the WHO/GARD model*.. Multidiscip Respir Med.

[3] Quanjer PH, Stanojevic S, Cole TJ, Baur X, Hall GL, Culver BH, ERS Global Lung Function Initiative (2012). Multi-ethnic reference values for spirometry for the 3-95 years age range: the Global Lung Function 2012 equations.. Eur Respir J.

[4] Wain LV, Shrine N, Miller S, Jackson VE, Ntalla I, Artigas MS (2015). Novel insights into the genetics of smoking behaviour, lung function, and chronic obstructive pulmonary disease (UK BiLEVE): a genetic association study in UK Biobank.. Lancet Respir Med.

[5] Jones PW, Quirk FH, Baveystock CM, Littlejohns P (1992). A self-complete measure for chronic airflow limitation - the St George’s Respiratory Questionnaire.. Am Rev Respir Dis.

[6] Jones PW, Harding G, Berry P, Wiklund I, Chen WH, Kline Leidy N (2009). Development and first validation of the COPD Assessment Test.. Eur Respir J.

[7] Beaton DE, Bombardier C, Guillemin F, Ferraz MB (2000). Guidelines for the process of cross-cultural adaptation of self-report measures.. Spine.

[8] Ställberg B, Nokela M, Ehrs PO, Hjemdal P, Jonsson EW (2009). Validation of the clinical COPD Questionnaire (CCQ) in primary care.. Health Qual Life Outcomes.

[9] Celli BR (2015). Recommendations for the early diagnosis of COPD: the AIMAR view.. Multidiscip Respir Med.

[10] Haroon S, Jordan R, Takwoingi Y, Adab P (2015). Diagnostic accuracy of screening tests for COPD: a systematic review and meta-analysis.. BMJ Open.

[11] Sarioglu N, Hismiogullari AA, Bilen C, Erel F (2016). Is the COPD assessment test (CAT) effective in demonstrating the systemic inflammation and other components in COPD?. Rev Port Pneumol.

[12] Wilcox TK, Chen WH, Howard KA, Wiklund I, Brooks J, Watkins ML (2013). Item selection, reliability and validity of the Shortness of Breath with Daily Activities (SOBDA) questionnaire: a new outcome measure for evaluating dyspnea in chronic obstructive pulmonary disease.. Health Qual Life Outcomes.

[13] Miravitlles M, García-Sidro P, Fernández-Nistal A, Buendía MJ, Espinosa de los Monteros MJ, Molina J (2013). Course of COPD assessment test (CAT) and clinical COPD questionnaire (CCQ) scores during recovery from exacerbations of chronic obstructive pulmonary disease.. Health Qual Life Outcomes.

[14] Miravitlles M, García-Sidro P, Fernández-Nistal A, Buendía MJ, Espinosa de Los Monteros MJ, Esquinas C (2015). The chronic obstructive pulmonary disease assessment test improves the predictive value of previous exacerbations for poor outcomes in COPD.. Int J Chron Obstruct Pulmon Dis.

[15] Sundh J, Janson C, Lisspers K, Montgomery S, Ställberg B (2012). Clinical COPD Questionnaire score (CCQ) and mortality.. Int J Chron Obstruct Pulmon Dis.

