# Supplementary material for: Development and the initial validation of a new self-administered questionnaire for an early detection of health status changes in smokers at risk for chronic obstructive pulmonary disease (MARKO questionnaire)
Source: Croat Med J. 2016 Oct;57(5):425–33. doi: 10.3325/cmj.2016.57.425 (PMC5141460; doi:10.3325/cmj.2016.57.425)
Supplement: Supplementary Table 1 [file CroatMedJ_57_s005.pdf]

MARKO
Screening Questionnaire

Identification: ________________________ Date of birth (MM/YYYY):__________ Sex: M W

Instructions

This questionnaire will give us the insight into your health and physical status and your breathing problems. Fill-in the questionnaire by encircling the letter in front of only one answer for each question that describes best your status.

Questions

1. Have you been coughing during preceding **3 months**?
   a) No
   b) Yes, but only during cold or respiratory infection
   c) Yes, 2 or 3 times each month
   d) Yes, many times each month
   e) Almost every day

2. Have you expectorated during preceding **3 months**?
   a) No
   b) Yes, but only during cold or respiratory infection
   c) Yes, 2 or 3 times each month
   d) Yes, many times each month
   e) Almost every day

3. Have you experienced being breathless during preceding **3 months**?
   a) No
   b) Yes, but only during cold or respiratory infection
   c) Yes, 2 or 3 times each month
   d) Yes, many times each month
   e) Almost every day

4. Have you had a severe cold with cough or bronchitis during preceding **12 months**?
   a) No
   b) 1 to 2 times
   c) 3 to 4 times
   d) 5 or more times

5. Have you used antibiotics for a severe cold with cough or bronchitis during preceding **12 months**?
   a) No
   b) 1 to 2 times
   c) 3 to 4 times
   d) 5 or more times

6. How do you assess you breathing?
   a) No problems whatsoever.
   b) A little problems with breathing.
   c) Moderate problems with breathing.
   d) A lot of problems with breathing.

Do you fill breathless during these activities:

7. Sitting, lying down, rest
   a) No breathlessness
   b) Mildly breathless
   c) Moderately breathless
   d) Severely breathless

8. Everyday activities like dressing up
   a) No breathlessness
   b) Mildly breathless
   c) Moderately breathless
   d) Severely breathless

9. Walking on the same level
   a) No breathlessness
   b) Mildly breathless
   c) Moderately breathless
   d) Severely breathless

10. Climbing to the first floor level
   a) No breathlessness
   b) Mildly breathless
   c) Moderately breathless
   d) Severely breathless

Questions

11. Climbing uphill
    a) No breathlessness
    b) Mildly breathless
    c) Moderately breathless
    d) Severely breathless

12. Strenuous physical activity
    a) No breathlessness
    b) Mildly breathless
    c) Moderately breathless
    d) Severely breathless

13. Do your problems with breathing interfere with activities that you like?
    a) No
    b) Yes, with one or two activities
    c) Yes, with several activities
    d) Yes, with all activities

14. When compared with most of your peers, how do you tolerate mild exertion (e.g. walking)?
    a) Better or comparable
    b) Somewhat worse than most
    c) Significantly worse than most
    d) Much worse than most

15. When compared with most of your peers, how do you tolerate moderate exertion (e.g. sports activities)?
    a) Better or comparable
    b) Somewhat worse than most
    c) Significantly worse than most
    d) Much worse than most

16. When compared with most of your peers, how fast you become tired?
    a) Slower or comparable
    b) Somewhat faster than most
    c) Significantly faster than most
    d) Much faster than most

17. How do you rate your health?
    a) Excellent
    b) Very good
    c) Satisfactory
    d) Unsatisfactory

18. How do you rate you breathing?
    a) Excellent
    b) Very good
    c) Satisfactory
    d) Unsatisfactory
